# Structural modelling of the lumenal domain of human GPAA1, the metallo-peptide synthetase subunit of the transamidase complex, reveals zinc-binding mode and two flaps surrounding the active site

**DOI:** 10.1186/s13062-020-00266-3

**Published:** 2020-09-29

**Authors:** Chinh Tran-To Su, Swati Sinha, Birgit Eisenhaber, Frank Eisenhaber

**Affiliations:** 1 Bioinformatics Institute, Agency for Science, Technology and Research (A*STAR), 30 Biopolis Street, # 07-01, Matrix, Singapore, 138671 Singapore; 2 School of Biological Sciences, Nanyang Technological University, 60 Nanyang Drive, Singapore, 637551 Singapore

**Keywords:** GPI lipid anchoring, Transamidase, GPAA1, GAA1, M28-type metallo-peptide-synthetase, 3D structural modelling, Molecular dynamics simulation, Phylogenetic tree

## Abstract

**Background:**

The transamidase complex is a molecular machine in the endoplasmic reticulum of eukaryotes that attaches a glycosylphosphatidylinositol (GPI) lipid anchor to substrate proteins after cleaving a C-terminal propeptide with a defined sequence signal. Its five subunits are very hydrophobic; thus, solubility, heterologous expression and complex reconstruction are difficult. Therefore, theoretical approaches are currently the main source of insight into details of 3D structure and of the catalytic process.

**Results:**

In this work, we generated model 3D structures of the lumenal domain of human GPAA1, the M28-type metallo-peptide-synthetase subunit of the transamidase, including zinc ion and model substrate positions. In comparative molecular dynamics (MD) simulations of M28-type structures and our GPAA1 models, we estimated the metal ion binding energies with evolutionary conserved amino acid residues in the catalytic cleft. We find that canonical zinc binding sites 2 and 3 are strongest binders for Zn1 and, where a second zinc is available, sites 2 and 4 for Zn2. Zinc interaction of site 5 with Zn1 enhances upon substrate binding in structures with only one zinc. Whereas a previously studied glutaminyl cyclase structure, the best known homologue to GPAA1, binds only one zinc ion at the catalytic site, GPAA1 can sterically accommodate two. The M28-type metallopeptidases segregate into two independent branches with regard to one/two zinc ion binding modality in a phylogenetic tree where the GPAA1 family is closer to the joint origin of both groups. For GPAA1 models, MD studies revealed two large loops (flaps) surrounding the active site being involved in an anti-correlated, breathing-like dynamics.

**Conclusions:**

In the light of combined sequence-analytic and phylogenetic arguments as well as 3D structural modelling results, GPAA1 is most likely a single zinc ion metallopeptidase. Two large flaps environ the catalytic site restricting access to large substrates.

**Reviewers:**

This article was reviewed by Thomas Dandekar (MD) and Michael Gromiha.

## Taxonomic abbreviations

Ab *Aneurinibacillus sp. AM-1*

Bs *Bacillus subtilis*

Bt *Bacteroides thetaiotaomicron*

Bv *Bacteroides vulgatus*

Ce *Caenorhabditis elegans*

Dm *Drosophila melanogaster*

Hs *Homo sapiens*

Is *Ixodes scapularis*

Lp *Legionella pneumophila*

Mm *Mus musculus*

Pd *Parabacteroides distasonis*

Pf *Plasmodium falciparum*

Pg *Porphyromonas gingivalis*

Sa *Shewanella amazonensis*

Sc *Saccharomyces cerevisiae*

Sg *Streptomyces griseus*

Vp *Vibrio proteolyticus*

## Background

As alternative to transmembrane regions, eukaryote proteins can be attached to the outer leaflet of the plasmalemma via a glycosylphosphatidylinositol (GPI) lipid anchor [1–4]. The molecular machine behind this reaction, the transamidase complex, recognizes substrate proteins with a four-partite C-terminal sequence signal [5–10] within the endoplasmic reticulum. A two-step reaction follows that involves (i) the cleavage of the C-terminal propeptide with the subsequent emergence of a new substrate protein C-terminus called ω-site and (ii) the formation of a peptide bond between the ω-site and an ethanolamine unit at the pre-synthesized GPI lipid anchor. Mutations in enzymes and auxiliary proteins of the GPI lipid anchor pathway are causative for a variety of human pathologies [1, 4, 11, 12].

The GPI lipid anchor pathway was discovered more than three decades ago. It was extensively studied in model organisms (yeast, trypanosomal and mouse/human systems) [1, 4, 13]. Yet, structural and functional details of the transamidase complex as well as of the catalytic process remain insufficiently understood as heterologous expression of its hydrophobic subunits and complex reconstruction attempts encounter experimental difficulties [14–17]. Therefore, theoretical efforts based on biomolecular sequence analysis and 3D structural modelling are an important source of insight. The human transamidase complex consists of five subunits (two proteolytic enzymes and three auxiliary proteins), each having one or multiple transmembrane regions and lumenal segments/domains apparently critical for function:

1. PIG-K (Gpi8p in yeast) is a C13-clade cysteine protease with structural similarity to caspases [1, 4]. This enzyme cleaves the C-terminal propeptide even without the presence of a GPI lipid anchor [18–21].
2. PIG-T (GPI16p in yeast) is predicted by sequence homology to form an unusual, open-ring C-terminal β-propeller structure with an additional α-helical N-terminal hook that can embrace another interacting, most likely protease subunit [1]. As a disulphide bond covalently links PIG-T (via Cys92) to PIG-K (via Cys182) [22], it is reasonable to conclude that PIG-T sits in front of PIG-K’s active site and regulates the access of substrates similar to homologous cases with the same type of β-propeller [1].
3. GPAA1 (GAA1 in yeast) is a M28-type metallo-peptidase/metallo-peptide-synthetase that catalyzes the formation of the peptide bond between the ω-site and the GPI lipid anchor’s ethanolamine unit [23, 24].
4. PIG-U (CDC91/GAB1 in yeast) [25], a protein with 10 transmembrane regions interconnected by loops, has the most likely function of shuttling the GPI lipid anchor and presenting it in a productive conformation to the transamidase complex and especially to GPAA1 [26].
5. PIG-S (GP17p in yeast), the smallest unit, remains functionally uncharacterized at the molecular function level [15, 17, 27].

**Fig. 1:**
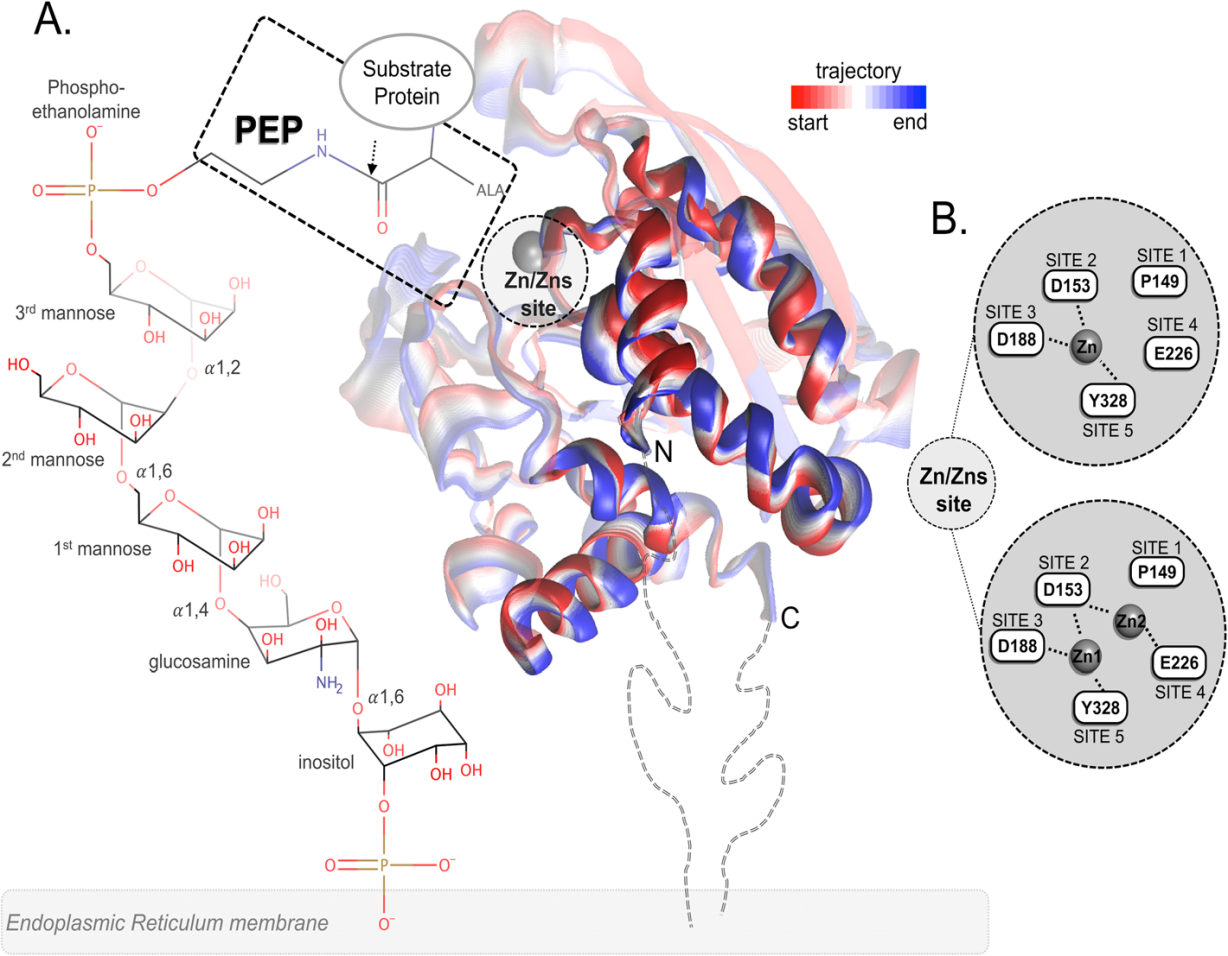
The GPAA1 model with one zinc ion and the substrate analogue. In this illustration, we show the 3D structural model of the lumenal domain of human GPAA1 resulting from the homology modelling effort together with (**a**) a scheme of the GPI lipid anchor at the endoplasmic reticulum (lipid part is omitted) as its terminal phospho-ethanolamine group reaches into the catalytic cleft where it comes into contact with zinc ion and the ω-site (the C-terminus) of the substrate protein pre-processed by PIG-K. The colour code (red/blue) of the model indicates the time-dependent position of the secondary structural elements along a molecular dynamics trajectory. Visibly, the *α* / *β* hydrolase fold, consisting of 8 strands and 7 helices, was maintained during the simulation. In section (**b**) of the figure, we highlight the five residues equivalent to the canonical Zn-binding sites typical for M28-type enzymes for the cases of one and/or two Zn ions. Dashed lines at the GPAA1 model N- and C-termini indicate that the lumenal domain is only a part of GPAA1 structure with additional loops in the endoplasmic reticulum lumen or cytoplasm and transmembrane regions residing in the endoplasmic reticulum membrane

This work is dedicated to the 3D structural modelling of human GPAA1 and the phylogenetic analysis of the GPAA1/GAA1 family in context with M28-type enzymes. The GPAA1 structure is most similar to the M28-type glutaminyl cyclase family that binds one zinc ion whereas many other M28-type enzymes bind two [23]. One Zn ion is commonly bound at sites 2, 3, and 5 and a possibly additional one at sites 1, 2, and 4 in the two zincs bound state [28–32] (see Fig. 1 in this work and Figure 1 in reference [23], Table 1). Here, we do not only provide model structures with atomic coordinates including metal ion and model substrate positions but we also explore the loop dynamics and the energetics of Zn ion binding by GPAA1 in a comparative molecular dynamics study and relate the results to phylogenetic analysis of Zn ion binding among M28-type enzymes. One of the surprising outcomes is that, upon substrate binding, the GPAA1 structure can accommodate even two Zn ions in the catalytic cleft whereas the glutaminyl cyclases exemplified by the 3D structure 4f9u [30] cannot.

**Table 1:** Summary of Zn-binding sites of structures used in the 3D structure modelling in this study

Structure	Site 1	Site 2	Site 3	Site 4	Site 5	Coordinated metal ions	Substrate	Ref.
4f9u	–	Asp^99^	Glu^139^	–	His^265^	Zn	SUB1	[30]
	His^82^	–	–	Asp^186^		no 2nd ion		
4fwu	–	Asp^99^	Glu^139^	–	His^265^	Zn	no substrate	[31]
	His^82^	–	–	Asp^186^	–	no 2nd ion		
1f2o		Asp^97^	Glu^132^	–	His^247^	Zn1	SUB2	[32]
	His^85^	Asp^97^	–	Asp^160^	–	Zn2		
1amp		Asp^117^	Glu^152^	–	His^256^	Zn1	No substrate	[28]
	His^97^	Asp^117^	–	Asp^179^	–	Zn2		
GAA1^Zn^	–	Asp^153^	Asp^188^	–	Tyr^358^	Zn	PEP or SUB1	(model / this work)
	*Pro^149^		–	Glu^226^	–	no 2nd ion		
GAA1^Zn1Zn2^	–	Asp^153^	Asp^188^	–	Tyr^358^	Zn1	PEP or SUB2	(model / this work)
	*Pro^149^	Asp^153^	–	Glu^226^	–	Zn2		

This table lists all the amino acid residues involved in the metal ion binding as well as the type of model substrates in the X-ray crystallographic 3D (including references) and model structures used in this work. Residue numbering follows the nomenclature in the published crystal structures and, for GPAA1, in UniProt sequence entry O43292. SUB1 stands for 1-(3,4-dimethoxyphenyl)-3-[3-(1H-imidazol-1-yl)propyl]-thiourea [30] and SUB2 is the label for L-leucine [32]. PEP is described in the text and in Fig. 1

*Proline 84 (site 1) does not bind to a Zn ion

## Results

### Homology modelling of the lumenal domain of the human GPAA1 protein

The programs I-TASSER [33, 34] and MODELLER [35, 36] were utilized to construct homology-based models of the lumenal segment of human GPAA1 (UniProt: O43292). The X-ray crystal structure of glutaminyl cyclase (PDB ID:4f9u, chain A [30]; a member of the M28 metallo-peptidases) was applied as the single template. To note, 4f9u has the best segment coverage following a previously published multiple sequence alignment (see Figure 1 in reference [23]). This alignment of GPAA1 (residues 66–348) and 4f9u (residues 4–298) was also used as an input to the programs to guide the modeling process.

Consistently in both modeling efforts using I-TASSER and MODELLER, the GPAA1 structural model exhibits an α/β hydrolase fold [23], comprising 8 strands and 7 helices in a compact structure (Fig. 1). The RMSD between the I-TASSER and MODELLER generated static structures is 1.33 Å, excluding the loops that cause larger deviations. Significant differences were observed at the loop 276–299 that is absent in the M28-type enzymes. Only the I-TASSER model was found to contain the expected additional small helix α_x_ (see Supplementary Figure S1 and Figure 1 in reference [23]). As the MODELLER version of the model does not comprise the extra helix in this region, we suggest that the I-TASSER result is the possibly better model for further analyses. Therefore, subsequently, we refer to the I-TASSER-generated structure if not mentioned otherwise.

### Insertion of Zn ions and of a tentative peptide (PEP) as substrate analogue

Because of the homology between the lumenal domain and the Zn-bound M28 peptidase family [23], we modeled the presence of zinc ion(s) and of a substrate analogue including a peptide bond into the catalytic cleft of the GPAA1 structural model. Sequence alignment of the five possible Zn-binding sites (see Figure 1 in reference [23] and Table 1) shows that, except for site 1 and 5, protein sequences of known 3D structures share similar physicochemical properties at sites 2, 3, and 4, e.g. bearing aspartate (D) or glutamate (E) that are canonical Zn-binding residues. While the reference structure 4f9u (and also all other 144 Zn(s)-bound structures in the M28 family metallo-peptide synthetase, extracted from Protein Data Bank and tabulated in Supplementary Table S1), contain histidine residues at both sites 1 and 5, the human GPAA1 sequence holds proline (P149, a residue unable to interact with a metal ion) and tyrosine (Y328) at sites 1 and 5, respectively. Notably, tyrosine is known to coordinate metal ions [37, 38].

Further in this text, GPAA1^Zn^ stands for a single Zn-binding GPAA1 model as in 4f9u/4fwu. We found that it is sterically possible to place yet another zinc ion into the model structure (as in the aminopeptidase structure 1f2o [29, 32], see also arguments below); thus, GPAA1^Zn1Zn2^ denotes the two Zn-binding model. The resulting GPAA1 model from I-TASSER was first subjected to the H++ server [39, 40] for hydrogen addition and protonated states estimation at lumenal pH 7.2 [41]. While maintaining the protonated state at the hydroxyl group of residue Y328 (site 5) [42], we removed hydrogens (if any) that were bound to OD2 atoms of D153 (site 2) and D188 (site 3) or to OD1 of D153 (site 2) and OE2 of E226 (site 4), respectively, due to their coordination with the zinc ion(s). Then, a non-bonded model was used in simulation of the Zn-coordinated GPAA1 binding sites using weak harmonic restraints (with force constants ~ 3 kcal/molÅ^− 1^).

**Fig. 2:**
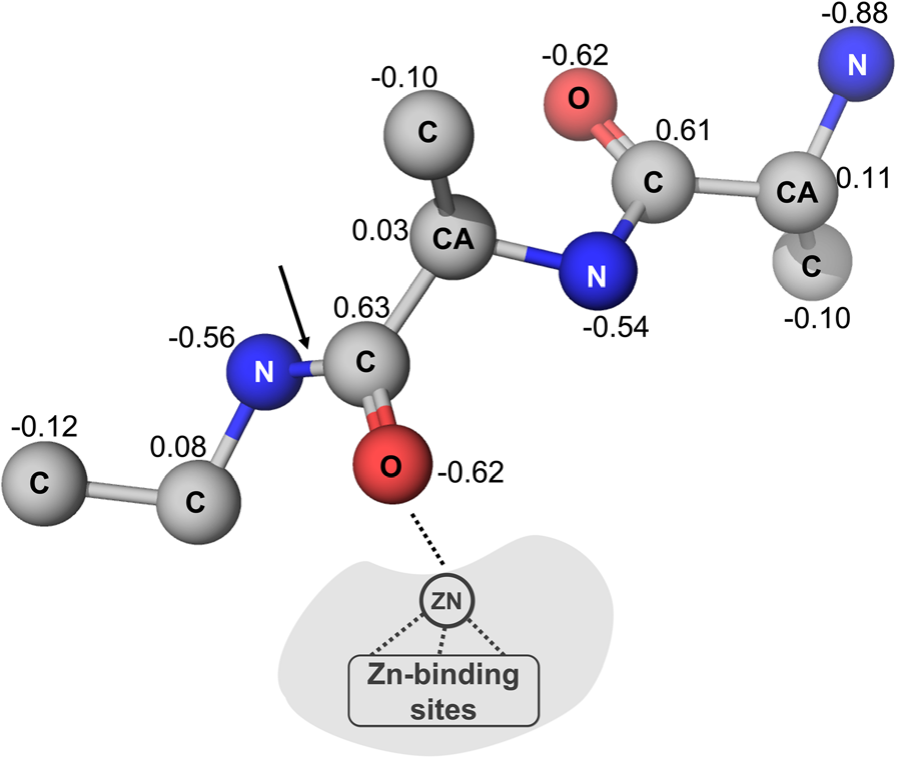
The model substrate for GPAA1 imitating a peptide bond between the ω-site and the ethanolamine. We show the structure of the tentative peptide (PEP: di-alanine ethylamine) together with its parameterization. The PEP is coordinated to one Zn via the carbonyl oxygen of the second alanine residue. The mimicked peptide bond formed between the ω-site and the phosphor-ethanolamine moiety is shown by a black arrow (compare also with Figure 2 in reference [23]). For clarity, hydrogen atoms are hidden, and partial charges are shown only for the heavy atoms

Also, we simulated the interaction of a tentative substrate peptide (di-alanine ethylamine, named “PEP” from now on) with human GPAA1 at the Zn binding sites. PEP was designed to mimic the product of the formed peptide bond between the ω-site of the potential substrate protein and the phospho-ethanolamine moiety at the GPI lipid anchor [1, 23]. For simplicity, an ethylamine group was attached to the second alanine of the PEP, which was then coordinated to one Zn ion via the carbonyl oxygen (Fig. 2; compare also with Figure 1 in [23]). The PEP was parameterized (assigning partial charges and atom types) using *antechamber* and GAFF force field implemented in the AMBER14 package [43, 44].

The resulting two models GPAA1^Zn^ and GPAA1^Zn1Zn2^ with their atomic coordinates are available as structure files in the supplementary material (files GPAA1_Zn_PEP.pdb and GPAA1_Zn1Zn2_PEP.pdb).

### Validation of the molecular dynamics simulation protocol

Before we can apply a molecular dynamics procedure to GPAA1 model structures, it is reasonable to elaborate a protocol that computes plausible values of desired parameters in the case of known M28-type structures. For this purpose, we first performed a molecular dynamics (MD) simulation using the AMBER14 package [45] with the force field ff14SB [46–48] to sample the conformational space of the two crystal structures 4f9u (one Zn-binding and model substrate 1-(3,4-dimethoxyphenyl)-3-[3-(1H-imidazol-1-yl)propyl]-thiourea referred to as SUB1) [30] and 1f2o (two Zn-binding and model substrate L-leucine referred to as SUB2) [29] as references. We chose to use ff14SB since it was shown to improve the helical content and side chain rotamer distributions in simulating biomolecules such as proteins [49].

The system was initially relaxed with 5000-step minimization (using steepest descent followed by conjugate gradient algorithms) and heated in gradual thermal baths from 0 K–100 K and then from 100 K–300 K in constant volume and pressure, respectively. Next, the system was equilibrated and used in the production processes applying explicit solvent model in triplicate repetitions of trajectories (3 × 300 ns). The simulations were carried out by assigning random velocities to the atoms constrained by the Langevin temperature equilibration scheme to stabilize the systems at 300 K at time steps of 2 fs. Analyses were performed on the portions of each trajectory where the simulations obtained stable, similar fluctuations of the five canonical zinc binding sites in comparison among the triplicates. Quantitatively speaking, we required stable backbone root mean squared deviation (RMSD ≤2.5 Å) of the five canonical Zn-binding site residues (Supplementary Figure S2). It was confirmed by the specific analyses on the reference structures that the simulation protocol reproduced the substrate positions (SUB1 and SUB2) in the Zn(s)-binding regions (e.g. in ~ 4 Å proximity compared to those of the starting structure) in the resulting ensembles of 4f9u (57%) and 1f2o (100%).

Then, the same protocol was applied to our 3D structural model of the lumenal domain of human GPAA1 and the two substrates SUB1 and SUB2. In both cases of the GPAA1^Zn^ and the GPAA1^Zn1Zn2^ models, the procedure resulted in outputting 54% of GPAA1^Zn-SUB1^ and 93% of GPAA1^Zn1Zn2-SUB2^ ensembles with similar proximity (~ 4 Å) of the substrate positions in the Zn(s) binding regions compared to their reference structures, respectively.

Therefore, the simulation protocol was applied to sample the conformational space of the GPAA1^Zn^ and GPAA1^Zn1Zn2^ models with the tentative peptide PEP. Also in these cases, the molecular simulations show compactness of packing as well as maintenance of secondary structure elements and hydrolase fold along the whole trajectory (Supplementary Figure S3).

### Structural models of the lumenal domain of human GPAA1 can accommodate either one or two zinc ions

Geometrically, the five residues of the active sites of both the M28 families (see Table 1), cyclotransferases (binding one zinc ion) and aminopeptidases/carboxypeptidases (binding two zinc ions), are in similar proximities. For instance, the distance between site 1 and Zn (or Zn1 in the case of two zincs) and between site 4 and Zn (or Zn1) are ~ 4.6 Å and ~ 5.9 Å, respectively (see the schematic active sites in Fig. 1b for the residue locations). With a Zn-Zn distance of 3.5 Å estimated in the aminopeptidase (e.g., in 1amp), this argument alone suggests that both the M28 family proteins should be able to sterically accommodate two zinc atoms in their active sites.

**Fig. 3:**
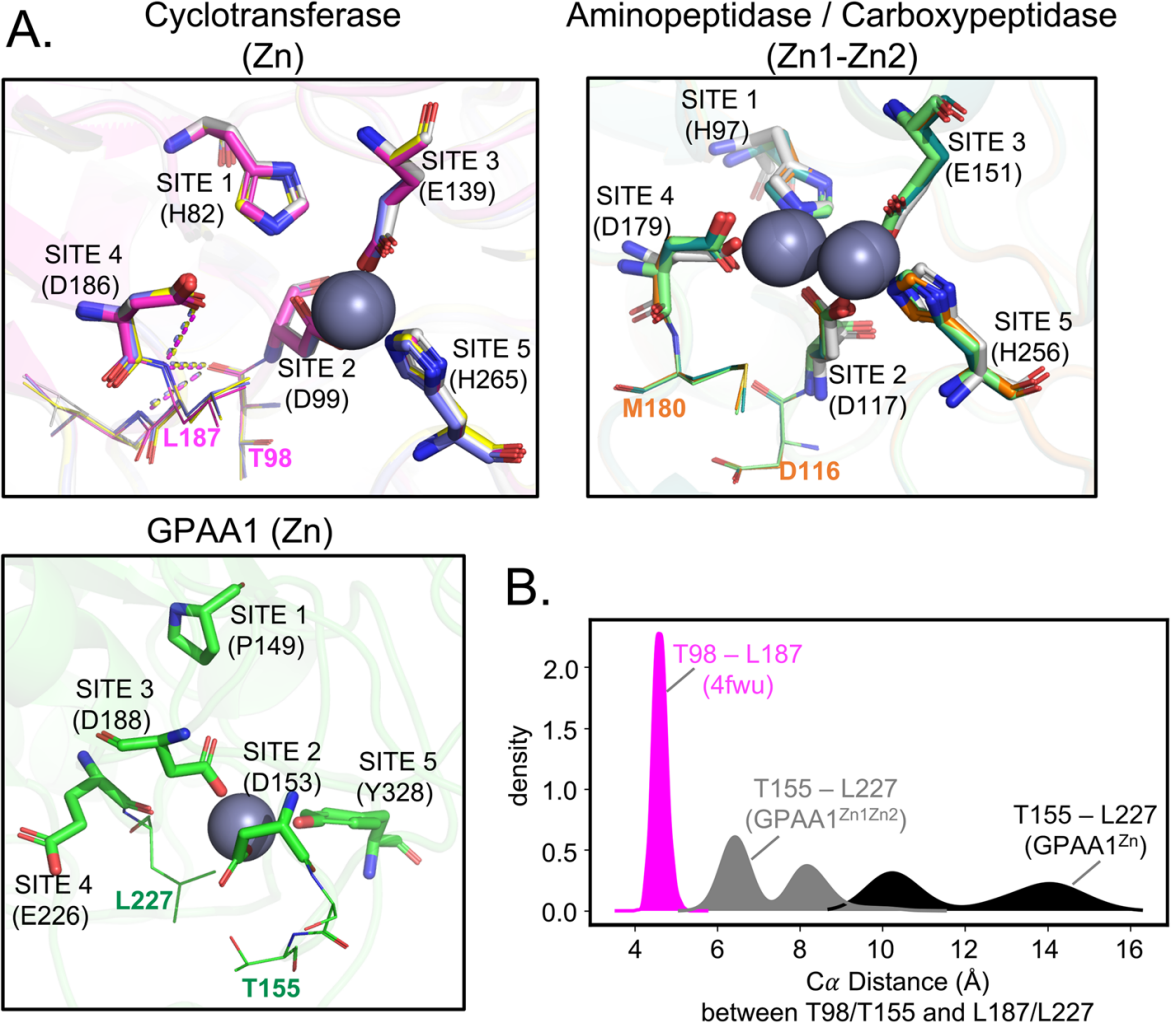
Comparison of zinc ion binding regions in M28-type metallo-proteases and in human GPAA1 model structures. **a** We present the canonical Zn-binding sites (in sticks) of a few one zinc-bound cyclotransferase proteins (4fwu, 4mhn, 4yu9, 3pbe; top left) and of a few two zinc-bound aminopeptidase/carboxypeptidase proteins (1amp, 1rtq, 2anp, 2c6g; top right). The labels and numbering are shown in accordance with the reference structures 4fwu (magenta; T98 and L187) and 1amp (orange, D116 and M180), respectively. The interactions between residues T98 and L187 (in lines) adjacent to site 2 and site 4 in the one zinc-bound cyclotransferase are represented as dashes. The five canonical active site residues of the GPAA1^Zn^ model (bottom left) are shown in green together with the nearby residues T155 and L227. **b** The distribution of the corresponding distances between the two residues during molecular dynamics trajectories are shown in magenta (T98 and L187 for 4fwu), black (T155 and L227 for GPAA1^Zn^), and in gray (T155 and L227 for GPAA1^Zn1Zn2^)

Yet, in the case of one Zn-bound structures (crystal structures of the cyclotransferase family, including the reference 4fwu [31]), it is noticed that site 2 (D99) and site 4 (D186) are bridged by a bifurcated hydrogen bond network between two adjacent residues T98 and L187, respectively (Fig. 3a, left). This observation is affirmed by a previous study that strong bifurcated hydrogen bonds can be formed by threonine or serine due to over-coordination between donor and acceptor groups in protein side chains [50]. One of the bifurcated hydrogen bonds occurs directly at the oxygen atom of the site 4 (D186) where, otherwise, it would be coordinated with a zinc atom (Zn2) in the case of aminopeptidases or carboxypeptidases.

However, these bifurcated hydrogen bonds are absent in aminopeptidases and carboxypeptidase that bind 2 zinc ions (Fig. 3a, right). The GPAA1^Zn^ model structure, bearing a large distance of ~ 6.3 Å between its site 4 and Zn1, can thus sterically accommodate another zinc atom in the Zn-binding cleft as in two zinc ion M28-type structures. At the same time, we do not find a pair of residues in the GPAA1^Zn^ model interacting in a similar way as T98/L187 in 4fwu. For example T155 and L227, a pair of possibly corresponding residues (Fig. 3a bottom), are located distantly and, during the simulation, they remain apart and no contacts were found between them (Fig. 3b). Thus, space for additional molecular entities is available. Therefore, we conclude that the GPAA1^Zn^ model is able to spatially accommodate another metal ion, e.g. a second zinc ion that could coordinate and bridge site 2 and site 4 in this region as in the GPAA1^Zn1Zn2^ model generated in this work. Independent molecular dynamics simulations of the GPAA1^Zn1Zn2^ 3D model showed that, in the presence of two zinc ions, the distance between the two residues T155 and L227 gets diminished but not to the range of T98/L187 in 4fwu (Fig. 3b). Notably, the second zinc ion remained stably fixated. Therefore, it can be speculated that the GPAA1 structure might alternatively adapt to / switch between single or duo zinc ion binding modes, models of which are generated in this work (see also arguments with regard to energetics of zinc ion binding further down).

### Dynamics of GPAA1 structures in the absence and presence of different substrates

For estimating the various sites’ contributions to the Zn-binding energies, we used the mmPBSA method [51] implemented in the Amber package. The energy decomposition analysis was performed using the generalized Born solvent model with ionic strength of 0.1 nM, with the non-polar solvation free energy estimated and proportional to the solvent accessible area. Meanwhile, the 1–4 electrostatics and 1–4 van der Waals were calculated and included in the internal (together with bond, angle, and dihedral) energies.

**Fig. 4:**
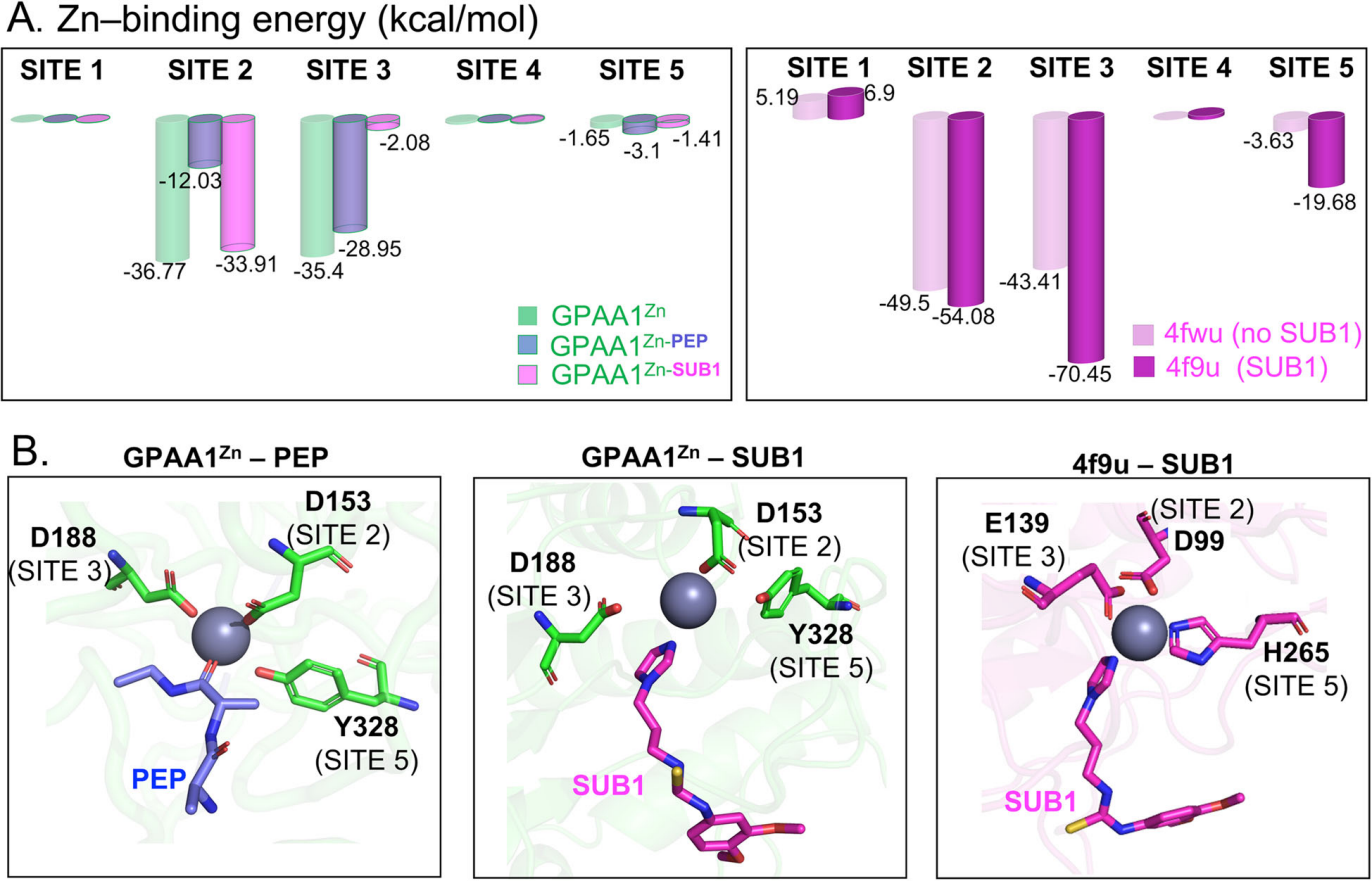
Energetics in single zinc-binding 3D structures. Analysis of molecular dynamics trajectories generated for the GPAA1^Zn^ model that binds one zinc ion and for 4fwu/4f9u. **a** Zn-binding energies at the five canonical sites estimated in the absence and presence of different substrates (PEP and SUB1) as compared to reference structures 4fwu and 4f9u, respectively. **b** Structural presentation of the binding regions (transparent cartoon) highlighting the binding site residues of GPAA1^Zn^ (green), 4f9u (magenta), and their corresponding substrates PEP (purple sticks) and SUB1 (magenta sticks). For better visualization, nitrogen atoms are shown in blue and oxygen atoms are in red. To simplify, hydrogen atoms are not shown. The Zn ion is represented as a grey sphere

**Fig. 5:**
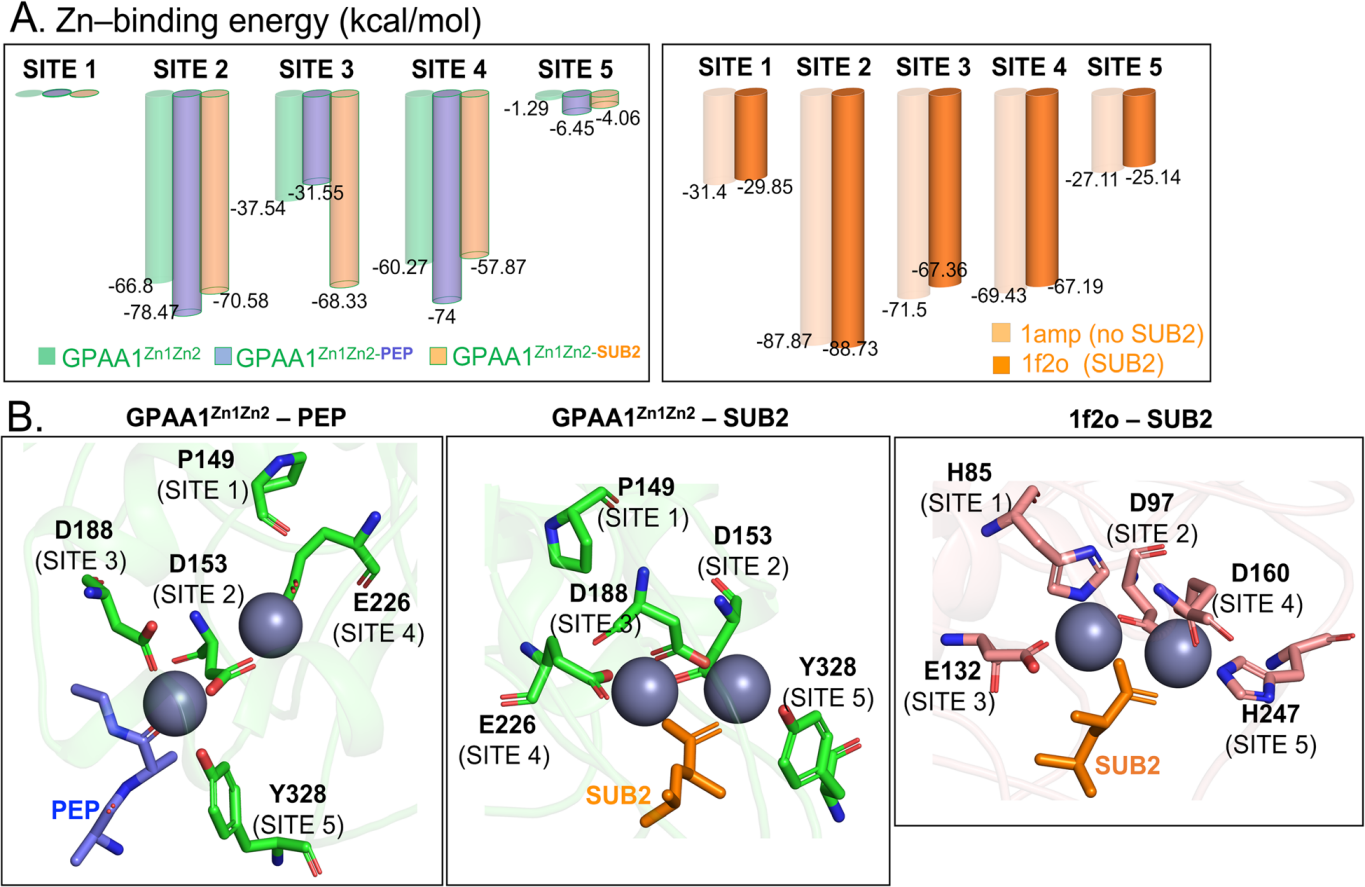
Energetics in duo zinc-binding 3D structures. Analysis of molecular dynamics trajectories generated for the GPAA1^Zn1Zn2^ model that binds two zinc ions and for 1amp/1f2o. **a** Binding energies at the five residual sites estimated in the absence and presence of different substrates PEP and SUB2 as compared to reference structures 1amp and 1f2o, respectively. **b** Structural presentation of the binding regions (transparent cartoon) highlighting the binding site residues of GPAA1^Zn1Zn2^ (green), 1f2o (orange), and their corresponding substrates PEP (purple sticks) and SUB2 (orange sticks). The nitrogen and oxygen atoms follow the same color scheme as in Fig. 4 with blue and red, respectively. To simplify, hydrogen atoms are not shown. The zinc ions are represented as grey spheres

For several M28-type family proteins, Zn(s)-bound structural forms are available in the absence of substrate, e.g. glutaminyl cyclase (4fwu [31]) with one zinc or aminopeptidase (1amp [28]) with two zinc ions). This observation suggests that Zn-binding events happen prior to substrate binding and result in a structural intermediate consisting only of the enzyme protein part with the metal ions. With molecular dynamics simulations without and with substrates, we estimated the energy contributions of zinc-binding residues in cases of one- and two-zinc metallopeptidase structures. We studied the following pairs of structures:

1. 4fwu and GPAA1^Zn^ – single zinc case both having no substrate (Fig. 4),
2. 4f9u (identical sequence with 4fwu) with SUB1 and GPAA1^Zn^ with PEP or SUB1 – single zinc case (Fig. 4),
3. 1amp and GPAA1^Zn1Zn2^ – duo zinc case both having no substrate (Fig. 5),
4. 1f2o with SUB2 and GPAA1^Zn1Zn2^ with PEP or SUB2 – duo zinc case (Fig. 5).

In both figures, panel A shows the per-residue energy contribution to zinc binding and panel B illustrates the position of zinc ions and of the canonical residues potentially interacting with the metal ions.

Throughout all sets of molecular dynamics simulations without model substrate (sets 1 and 3), we find that canonical site 5 tends to contribute the least to the zinc-protein interactions (Fig. 4a and 5a). Similarly, site 1 (where applicable) is also not a major contributor. The trends in the energy contribution of canonical sites 2, 3, and/or 4 to zinc ion binding for the GPAA1 models are markedly similar compared to those in the reference structures (4fwu and 1amp) showing considerably lower energies than site 5 in Zn(s) ions interactions. Therefore, GPAA1 lumenal domain structures could, indeed, structurally accommodate either one or two zinc atoms in its active sites.

In the case of the single Zn-bound complex 4fwu/4f9u, the presence of substrate (trajectory set 2) was found to amplify interactions between the zinc ion and the enzyme’s canonical site residues, especially remarkably for site 5 (Fig. 4). In the reference structure (4f9u, with the natural substrate SUB1), we observe a ~ 6 fold energy increase at site 5 (Fig. 4a, right panel). Likewise, in the GPAA1^Zn^ model, binding of the more natural model substrate PEP (but not of the substrate SUB1) results in stronger interaction (about 2x) between Zn and site 5 (Fig. 4a, left panel) mostly due to contributions of van der Waals and electrostatics potential energy (Supplementary Figure S4). One may conclude, therefore, that the strength of the interaction between Zn and site 5 depends on the substrate binding in the case of single zinc metallopeptidases (Fig. 4a, left panel). Depending on the substrate (the more natural PEP or SUB1 placed for comparison), we observe varying Zn-binding energies at canonical sites 2 and 3 (opposite trends) in the GPAA1^Zn^ model (Fig. 4a, left panel).

The energetics of zinc binding in the known duo zinc complexes (as exemplified by 1amp/1f2o, molecular dynamics trajectory sets 3 and 4) upon substrate binding is quite different (Fig. 5). Although we see again that canonical sites 1 and 5 are of less importance in the zinc coordination, there is not much change in the energy contributions for all five sites upon substrate SUB2-binding (Fig. 5a, right panel). In the case of the GPAA1^Zn1Zn2^ model (Fig. 5a, left panel), we find a trend for strengthened interaction of zinc with sites 2 and 4 (for PEP) and with site 3 (for SUB2). Remarkably, interaction with site 5 is drastically enhanced upon substrate binding (5x for PEP, 3x for SUB2) in complete contrast with computation results for 1amp/1f2o.

Since the GPAA1 structure reveals energetics of zinc binding most similar to those of the reference glutaminyl cyclase structures 4fwu/4f9u (especially with regard on the enhancement of site 5 interaction upon substrate binding), it is tempting to conclude that the GPAA1 lumenal domain has just one zinc ion for its catalytic function in GPI-attachment. As site 1 tends to be not of major significance, the change to proline (from histidine in most M28-type enzymes) in the case of human GPAA1 does not have a dramatic functional effect. Being not essential for catalysis, the results suggest that site 1 (proline) is probably serving another role in the GPAA1 structure and/or function.

### Observations of loop dynamics in the molecular simulations of GPAA1 3D models

**Fig. 6:**
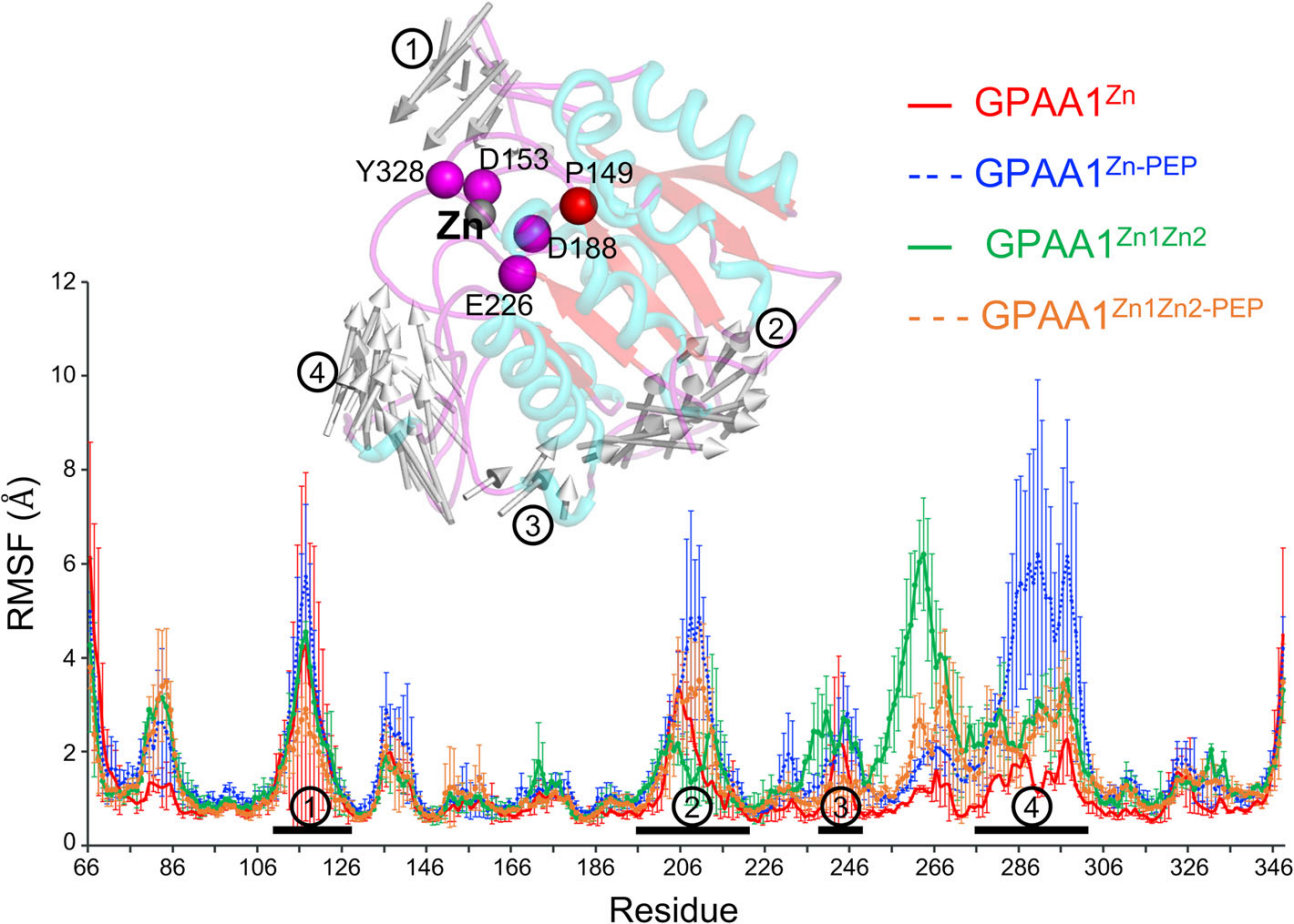
Positional fluctuation (RMSF) of the GPAA1 models with/without binding to PEP. The diagram in the lower part of the figure shows that four large loops have particularly extensive motions during molecular dynamics simulations regardless of the substrate and one/duo zinc ion binding modes. These loops are labelled as (1) residues 111–130, (2) residues 197–215, (3) residues 240–250, and (4) residues 276–299 including a small helix (훼_x_ as predicted in reference [23]). An illustration of the 4 loop motions (in the case of the GPAA1^Zn^ model) are shown on the top of the figure with grey arrows indicating the motion vectors, the length of which represents the magnitude of the movements. The GPAA1 structure is shown with helices in cyan, strands in red, and loops in magenta. The five Zn-binding residues are represented by their Cα atoms as coloured spheres. The zinc ion is presented as a grey sphere

There are four large loops in the structure of GPAA1 (see Fig. 6). GPAA1 sequences contain an insertion between strand β7 and helix α6 (see Figure 1 in reference [23]; residue D276 followed by sequence WTSLDGPLQGLQTLLLMVLRQASG in human GPAA1) that is absent in other sequences of the M28 family. This stretch forms a long loop (including a small additional helix) that is located at the opening of the GPAA1 active site cleft (loop 4 in Fig. 6). We observe during the molecular dynamics simulations of the GPAA1 models in both one and two zinc-bound cases that the loop 4 is preferentially in two conformational states, “***closed***” and “***open***” relative to the GPAA1 zinc location site. In particular, in the case of GPAA1^Zn^, the distances between the centre of mass of the loop and the Zn ion(s) are ~ 15.52 Å (***closed***) and ~ 24.87 Å (***open***). These distances are respectively ~ 21.64 Å (***closed***) and ~ 44.84 Å (***open***) in the case of GPAA1^Zn1Zn2^. For comparison, the diameter of an α-helix is ~ 12 Å.

Interestingly, this loop 4 was found to be coupled with the motion of another loop spatially near the GPAA1 zinc location site, the loop between strands β1 and β2 (see Figure 1 in reference [23], residues 118–130 with the THERYMVSGTNVY - in human GPAA1 sequence, loop 1 in Fig. 6). Noteworthy, this loop is also longer than homologous segments in other M28-type sequences (see the alignment of reference [23]). The two loop motions were found anti-correlated (calculated using Pearson correlation between the distance of the two loops to the zinc location site, resulting in the R_pearson_ = − 0.78, *p*-value << 0.0001 with 95% confidence interval), forming opposite direction flaps surrounding the GPAA1^Zn^ active site and exhibiting a breathing-like dynamics (Fig. 6). The two flaps move more flexibly in the presence of the substrate PEP (blue curve in Fig. 6). In the case of two Zn-bound GPAA1^Zn1Zn2^, the motions of the two flaps are found much more weakly and not anti-correlated (R_pearson_ = 0.5, p-value << 0.0001 with 95% confidence interval).

In both cases of Zn(s)-bound GPAA1 models, however, the motions of the two flaps do not affect the exposure of zinc ions at the active sites for small substrates such as the model substrate PEP. Therefore, it is inferred that the GPAA1 active site is mostly maintained and supported by the core scaffold of helices and strands also resembled in other M28-type family proteins. Yet, the real substrates of GPAA1 are the GPI lipid anchor precursor and the C-terminus of an eligible substrate protein, both being quite bulky entities. Thus, the movement of flaps consisting of loops 1 and 4 has a likely role in regulating their access to the active site and/or in accompanying the catalytic process of substrate binding and release.

Another characteristic, conserved motif in GPAA1 sequences (but not in other M28-type enzymes), the C-terminal end of strand β6, the N-terminal part of the following helix α5 and the loop in-between (see alignment in Figure 1 of [23]; sequence starting with E240 followed by GNLGXLPNLD in human GPAA1) is located distantly from the GPAA1 active site (actually, at the opposite side of the structure) and exhibits more restrained motions compared to the other loops in the GPAA1 (see loop 3 in Fig. 6). Therefore, we think that this segment is not involved in the substrate binding process of GPAA1 but has another significance, most likely in transamidase complex formation. The structural modelling results are coherent with experimental results of Vainauskas et al. [52], who demonstrated that mutation of the GLNG stretch to AAAA drastically weakens but not completely excludes interactions with other transamidase subunits.

### Comparison with the structural models of the lumenal domain of yeast GAA1 from Gamage et al. [14]

Our model structure has a radius of gyration R_g_ ~ 20.5 Å. This result is similar to the radius of gyration that we computed from the structure files of two yeast GAA1 models by Gamage et al. [14] supplied in their supplementary material, 20.1 Å (the one derived from 1RTQ [53, 54] with Rosetta [55]) and 20.8 Å (the model derived from 4f9u [30] with RaptorX [56, 57]) respectively (see Supplementary Fig.ure S5 for structural illustrations). The RMSDs of our structural model with those two from Gamage et al. are 5.28 Å and 4.98 Å, respectively. For comparison, the RMSD between the two Gamage et al. model structures is 2.9 Å. To note, GAA1 is the yeast homologue of human GPAA1 with ~ 36% sequence similarity in the lumenal domain part. The structural scaffolds of the three models are very similar (especially between their 4f9u-derived and our model; see Supplementary Figure S5); the variation comes overwhelmingly from the differently arranged loops. Only in our model, the presence of the Zn ion at the catalytic cleft is modelled and the additional small helix α_x_ is found located in the flexible loop (loop 4 in Fig. 6).

Structural alignment revealed that, instead of the loop 4 expected from secondary structure predictions (Figure 1 in [23]), the Rosetta model (black in Supplementary Figure S5) contains an additional long helix. This difference has likely functional consequences. In our GPAA1 model, loop 4 surrounds the Zn-binding cleft. The large surplus helix in the Rosetta model apparently diminishes polypeptide chain flexibility and changes modulation of substrate accessibility, which is, on the other hand, accommodated by the more flexible loop in our model.

### Structural analysis of the human equivalent of the yeast deletion mutant protein yGAA1^70–247^

Saw et al. [16] using SAXS studied the structure of a truncated version of GAA1, the yeast homologue of GPAA1. The measured radius of gyration R_g_ ~ 27 Å is significantly larger than that of the full-length model of the lumenal domain of human GPAA1 generated in this work (~ 20 Å). Analyzing our GPAA1 3D model, we wished to rationalize (i) why the truncated mutant forms a stable structure in solution and (ii) why the gyration radius of the mutant is so much larger.

**Fig. 7:**
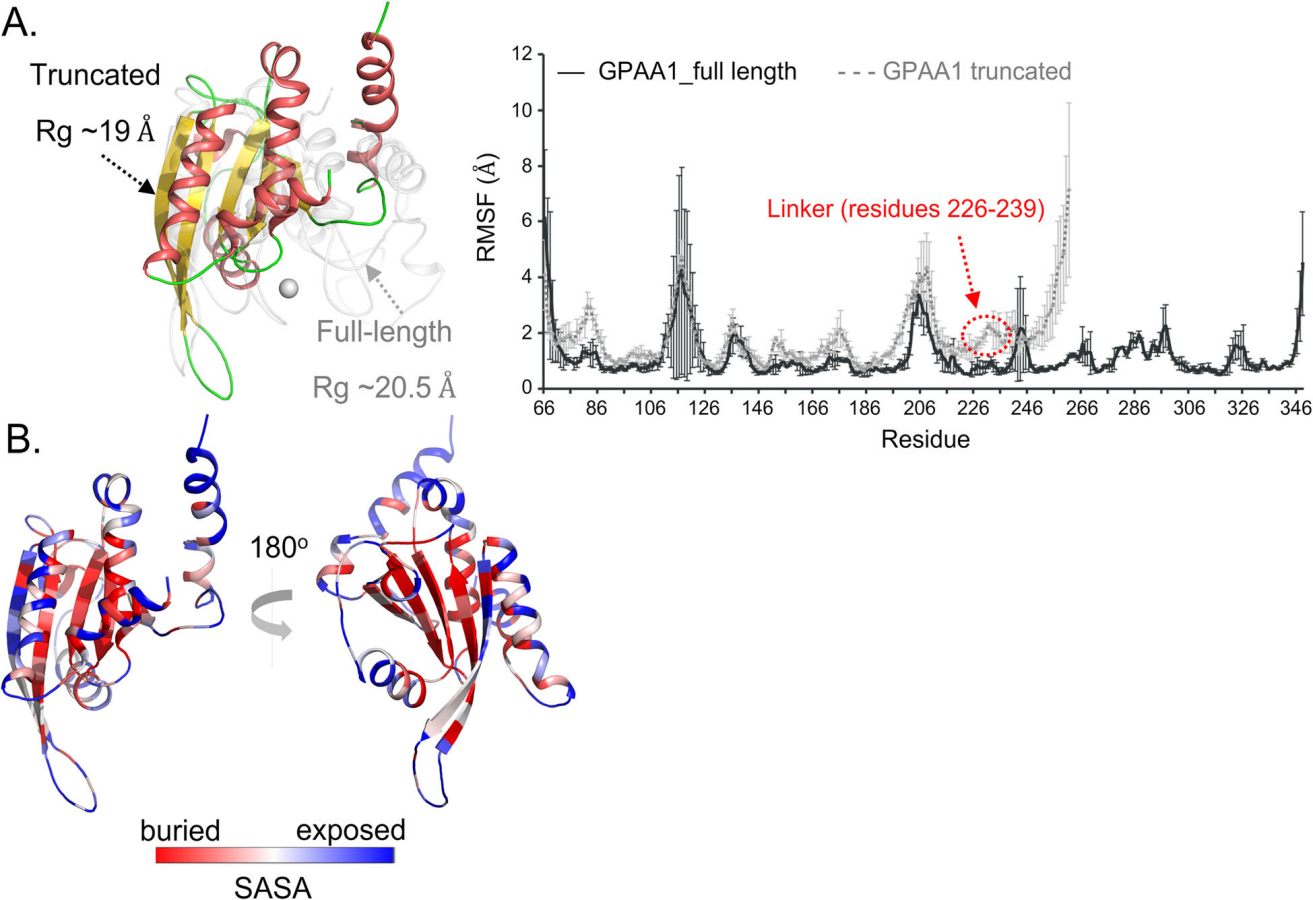
Analysis of the model of GPAA1^Zn^ truncated in the same way as the deletion mutant protein yGAA1^70–247^ from Saw et al. [16]. **a** At the left side of panel A, we show the model of the truncated structure (coloured) at the background of the full-length GPAA1^Zn^ model (grey). At the right side, we present a diagram illustrating and comparing the spatial fluctuations of residues during a molecular dynamics simulation for the truncated (grey) and the full-length (black) GPAA1^Zn^ model structure. Notably, the α/β fold remained stable along the MD trajectory. The linker (residues 226–239 in accordance with residue numbering in the GPAA1^Zn^ model) highlighted with a dashed circle that shows larger fluctuation compared to the corresponding region in the full length GPAA1 structure may allow more flexibility at the C-terminus of the truncated GPAA1. **b** The hydrophobic core was found buried in the truncated model, demonstrating that the truncated GPAA1 could avoid aggregating as it was observed experimentally by Saw et al. [16]

The mutant, most likely non-functional protein yGAA1^70–247^ lacks all secondary structural elements beyond the C-terminal side of helix α5 as well as the canonical zinc-binding site 5. Saw et al. [16] found yGAA1^70–247^ as a monomer in solution and its shape was best approximated by large elliptical volume (71 Å × 48 Å) that is connected via a short stalk (length 8 Å, average diameter 8 Å) to a smaller hook-like domain (8 Å × 35 Å). We observed a similar shape in our truncated atomistic model (Fig. 7) in which the N-terminal core scaffold of helices and strands is connected to the C-terminal helix α5 (with a length of ca. 20 residues and > 30 Å) via a flexible linker (residues 226–239 in human GPAA1). Indeed, the hydrophobic core was found buried in the truncated model and the fold remaining stable in a comparative molecular dynamics study with the full-length model (see Fig. 7), demonstrating that the truncated GPAA1 could avoid aggregating as was found in the experiments described by Saw et al. [16].

Due to the lack of structural constraints by the rest of the scaffold, i.e. residues 262–348 present in the full length GPAA1, the linker can exhibit more flexible motions (Fig. 7) as compared to that of the full length model, resulting in larger fluctuation of the C-terminal helix α5 in the truncated version. We suppose that this flexible linker and the dangling helix α5 are responsible for the more extended conformation of the truncated protein.

### Comparative phylogenetic analysis of the GPAA1 family and of M28-type sequences with known structures

An HHpred [58] search of the M28 peptidase (Pfam: PF04389) seed alignment was performed against the PDB sequence database (PDB_mmCIF70_27_Apr). In addition, 3D-structures belonging to M28 family were retrieved from the PDB database using searches for annotation/description line items. By combining the resulting hits from both these approaches and manually filtering to include only the proteins having an M28 domain, a comprehensive set of 145 PDB 3D-structures belonging to the M28 family was created (annotated as 42 aminopeptidases (AM), 66 carboxypeptidases (CP) and 37 cyclases/cyclotransferases (CT); Supplementary Table S1). The selected 3D-structures were grouped into classes of the corresponding UniProt protein sequences disregarding minor mutations, variations in substrates, etc. We found that all these structures map to just 20 unique UniProt sequences (see Table 2 for a set of representative structures annotated for zinc binding). To note, the example 3gux, although without description of its Zn ion binding status, was added as it is part of the alignment in Figure 1 of ref. [23].

**Table 2:** Number of experimentally proven zinc ions per protein molecule located in structures used in the phylogenetic tree analysis in this study

PDB ID	UniProt ID	Annotation	Organism	Number of bound Zn ions	Ref.
1AMP	Q01693	Leucyl aminopeptidase	*Vibrio proteolyticus (Vp)*	2	[28]
1TKJ	P80561	Aminopeptidase	*Streptomyces griseus (Sg)*	2	n/a
1Z8L	Q04609	Carboxypeptidase	*Homo sapiens (Hs)*	2	[59]
2AFO	Q16769	Cyclotransferase	*Homo sapiens (Hs)*	1	[60]
3FEC	Q9Y3Q0	Carboxypeptidase	*Homo sapiens (Hs)*	2	[61]
3GUX	A6KZZ2	Leucine aminopeptidase	*Bacteroides vulgatus (Bv)*	1	n/a
3IIB	A1S420	Aminopeptidase	*Shewanella amazonensis (Sa)*	2	n/a
3 PB4	Q9NXS2	Cyclotransferase	*Homo sapiens (Hs)*	1	[62]
3SI1	Q9CYK2	Cyclotransferase	*Mus musculus (Mm)*	1	[63]
3TC8	A6LHT4	Leucine aminopeptidase	*Parabacteroides distasonis (Pd)*	1	n/a
4F9U	Q9VRQ9	Cyclotransferase	*Drosophila melanogaster (Dm)*	1	[30]
4FAI	Q86PD7	Isoglutaminyl cyclase	*Drosophila melanogaster (Dm)*	1	[30]
4FUU	Q8A4P9	Leucine aminopeptidase	*Bacteroides thetaiotaomicron (Bt)*	1	n/a
4MHN	B7QK46	Cyclotransferase	*Ixodes scapularis (Is)*	1	n/a
4TWE	Q9UQQ1	Aminopeptidase	*Homo sapiens (Hs)*	2	[64]
5GNE	Q5ZZH8	Leucine aminopeptidase	*Legionella pneumophila (Lp)*	2	[65]
5IB9	A2V759	Aminopeptidase	*Aneurinibacillus sp. AM-1 (Ab)*	2	n/a
6ESL	Q5ZRR6	Aminopeptidase	*Legionella pneumophila (Lp)*	2	[66]
6HC6	P25152	Aminopeptidase	*Bacillus subtilis (Bs)*	2	n/a
6QQL	Q7MT37	Glutamine cyclotransferase-related protein	*Porphyromonas gingivalis (Pg)*	1	n/a

This table lists all the PDB structure codes with annotations, literature references (n/a - not applicable if none is available) and number of Zinc ions bound per protein molecule

A multiple sequence alignment of the selected 20 M28-type sequences with known structures, along with six GPAA1/GAA1 sequences, was created using inputs from HHpred [58] and MUSCLE [67, 68] and was subsequently adjusted manually. MEGA X [69] was used to infer the evolutionary history by using the Maximum Likelihood method and JTT matrix-based model with 1000 bootstrap replicates [70].

**Fig. 8:**
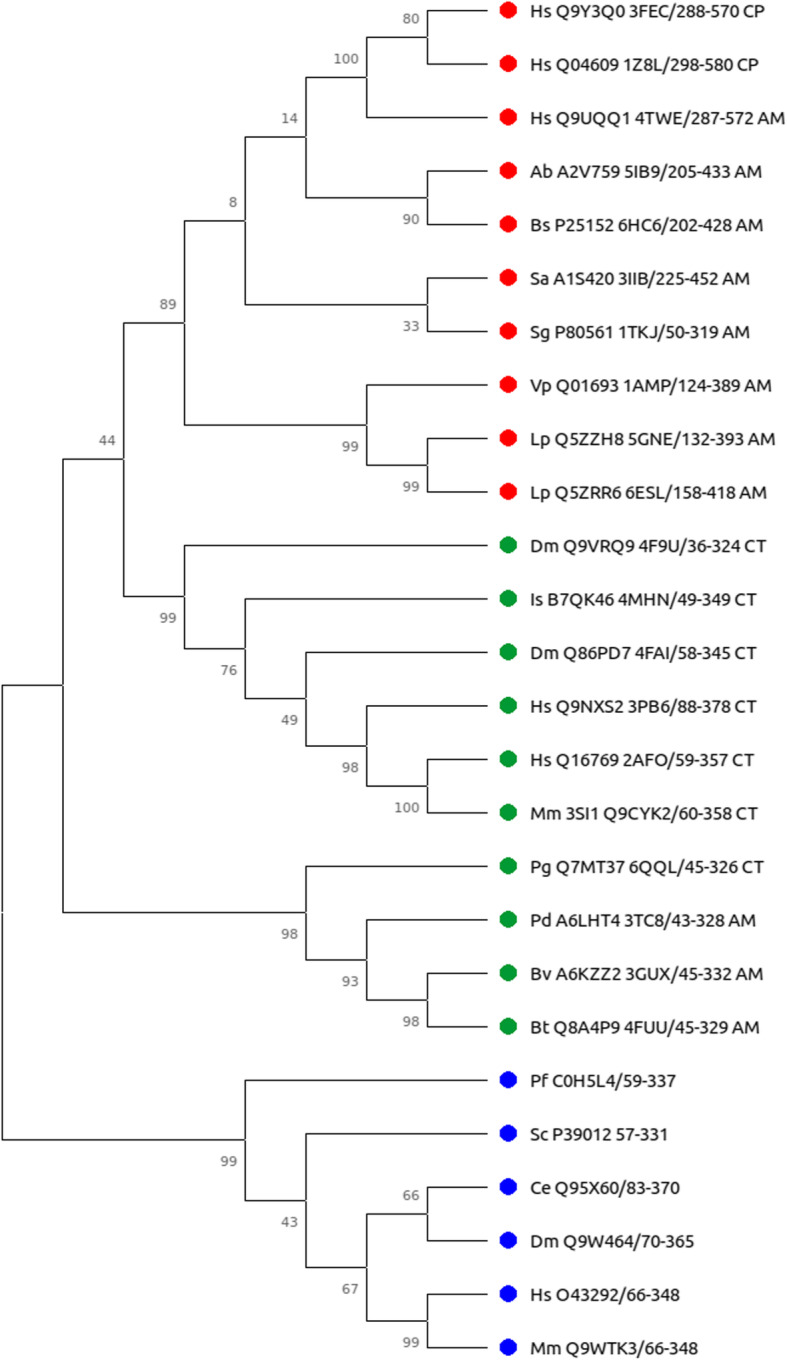
Phylogenetic tree of M28-type sequences with known structures and sequences of the GPAA1 family. The evolutionary history was inferred by using the Maximum Likelihood method and JTT matrix-based model [70]. The tree with the highest log likelihood (− 15093.41) is shown. The percentage of trees in which the associated taxa clustered together is shown next to the branches. Initial tree(s) for the heuristic search were obtained automatically by applying Neighbor-Join and BioNJ algorithms to a matrix of pairwise distances estimated using the JTT model, and then selecting the topology with superior log likelihood value. This analysis involved 26 amino acid sequences. Evolutionary analyses were conducted in MEGA X [69]. For each leave of the phylogenetic tree, we list (i) a color code, (ii) the taxonomic abbreviation, (iii) the accession number from the protein data bank RCSB or UniProt, (iv) the sequence segment from the original sequence used for the alignment behind the tree, (v) the sequence length of the sequence segment and (vi) the molecular function code (AM aminopeptidase, CP carboxypeptidase, CT cyclotransferase, CTI cyclotransferase type I, CTII cyclotransferase type II). The sequence groups presented in the phylogenetic tree are (i) known two zinc-binding 3D structures 3fec [61], 1zbl [59], 4twe [64], 5ib9, 6hc6, 3iib, 1tkj, 1amp [28], 5gne [65], and 6esl [66] color-coded red; (ii) known single zinc-binding 3D structures 49fu [30], 4mhn, 4fai [30], 3pb4 [62], 2afo [60], 3si1 [63], 6qql, 3tc8, and 4fuu color-coded green as well as (iii) six sequences from the GPAA1 family color-coded blue. We show also the position of 3gux in the tree although it is not known how many zinc ions it does bind in the catalytic cleft (we predict one) as it was part of the alignment in Figure 1 of reference [23]. To note, there is no scientific article available for structures without listed reference above

The phylogenetic tree as shown in Fig. 8 shows a clear dichotomy for structures reported as one (1Zn) and two (2Zn) Zn ion binding M28-type peptidase family members. The branch consisting of cyclotransferases/cyclases (marked with green circles) that bind a single zinc ion contain sequences belonging to arthropods (4mhn/B7QK46, 4f9u/Q9VRQ9, and 4fai/Q86PD7), bacteria (6qql/Q7MT37 – cyclotransferase type I, CTI) and mammals (3pb4/Q9NXS2, 2afo/Q16769, and 3si1/Q9CYK2 – cyclotransferases type II, CTII).

In addition, three examples annotated as leucine aminopeptidases (3tc8/A6LHT4, 3gux/A6KZZ2, and 4fuu/Q8A4P9) also cluster in the same clade. There is no publication available for any of these three structures. In order to verify whether this clustering is correct and to exclude possible annotation errors in the sequence database, we did a BLAST search for all the proteins without verifiable molecular function/enzymatic activity (via experimental evidence reported in the scientific literature) against the non-redundant protein sequence database. For example, 3tc8/A6LHT4 (leucine aminopeptidase) shows high identity to both arginyl aminopeptidase (WP_057326189.1, E-value = 0 and PI = 99.7%), and glutamine cyclotransferase (OKY96427.1, E-value = 0 and PI = 99.7%). Similarly, 3gux/A6KZZ2 (leucine aminopeptidase) hits the glutamine cyclotransferase (RHJ01572.1, E-value = 0 and PI = 99.7%) as the best hit. Furthermore, 4fuu/Q8A4P9 (leucine aminopeptidase) shares high sequence identity (> 99%) with both leucine aminopeptidase (CUP85417.1, E-value = 0, PI = 99.7%) and glutamine cyclotransferase-related protein (EFI02281.1, E-value = 0 and PI = 99.7%). Similar results were observed for 6qql/Q7MT37 (glutamine cyclotransferase). This suggests that some of the available annotations in the databases are dubious and, therefore, need experimental validation for better function assignment. This will add more information to the current pattern of leucine aminopeptidases clustering with cyclotransferases, as observed in the tree.

The other branch consists of members that bind two Zn ions (2Zn), marked with red circles, which include two carboxypeptidases (3fec/Q9Y3Q0 and 1z8l/Q04609) and eight aminopeptidases (5ib9/A2V759, 6hc6/P25152, 3fec/Q9UQQ1, 3iib/A1S420, 1tkj/P80561, 1amp/Q01693, 5gne/Q5ZZH8, and 6esl/Q5ZRR6). At the same time, GPAA1/GAA1 family members, marked with blue circles, cluster as an outgroup and, consequently, are directly related to the common ancestor of both 1Zn- and 2Zn-binding M28 structures. The protein 4f9u/Q9VRQ9 is closest to the tree origin among M28 type structures and, thus, not surprisingly the best/closest homologue. We also show the position of 3gux/A6KZZ2 in the tree although it is not known how many zinc ions it does bind in the catalytic cleft (we predict one) as it was part of the alignment in Figure 1 of reference [23].

## Discussion

As it was pointed out by Chevrier et al. [28, 71], M28-type enzymes fall into two classes given the number of zinc ions bound at the active site. It is possible that one Zn ion per active site only fulfils the catalytic function alone. In the case of two Zn ions, there can be one substrate processing site with one zinc ion (and the second zinc ion has an amplifying/regulating role) or both Zn ions are primary subsites for catalysis, possibly in an alternating order and/or in a pseudo-symmetric arrangement.

Structural modelling of the lumenal domain of human GPAA1 has shown that the intriguing opportunity of a second zinc transiently being involved in the catalytic process cannot be ruled out by structural exclusion arguments alone. This result implies two options.

(i) For catalysis, GPAA1 binds one zinc ion per active site.
(ii) GPAA1 also binds a second ion in an intermediary state (static binding is unlikely as site 1 residue is a proline that cannot bind a metal ion).

This observation is sharply contrasting with what we see in cyclotransferase structures such as 4f9u where placing a second metal ion in the active site is sterically impossible despite of all five canonical zinc-binding sites present. In molecular dynamics simulation of GPAA1^Zn1Zn2^, we find the second zinc stably bound in catalytic cleft; thus, the energetics of duo zinc ion positioning are tolerable. Therefore, we cannot absolutely exclude binding of a second zinc ion in an intermediary state at this point. Nevertheless, we tend to favor a single zinc active site for GPAA1 at this stage with the following arguments:

1. Sequence-analytic argument: The canonical zinc-binding site 1 is a proline in most GPAA1s (asparagine in Plasmodium and glutamine in worm) whereas it is a histidine in the overwhelming number of M28-type enzymes. Thus, any second metal ion could only be coordinated by two amino acid residues from GPAA1 instead of the canonical three (when a fourth valence could be occupied by the substrate).
2. 3D structural argument: The binding energies of the zinc ion in the single ion case for human GPAA1 resemble the situation for 4f9u and 4fwu (Fig. 4). In the case of substrate positioning, we observe the same enhancement of zinc interaction with site 5. There is no enhancement of zinc interaction with site 5 in M28-type structures with two metal ions in the catalytic cleft (Fig. 5).
3. Phylogenetic argument: Although the phylogenetic tree reveals that GPAA1s are related to the common predecessor of all M28-type sequences (Fig. 8), it is the 4f9u sequence that is closest to the origin of the M28-type structures’ phylogenetic tree. Thus, the common predecessor of all three sequence groups was most likely a single zinc ion enzyme.

There are major differences between the GPAA1/GAA1 family sequences and those of the M28 groups in some of the loop regions, especially with regard to the much longer loop 4 (see Fig. 6 for nomenclature). Together with another large loop 1, it forms a two-flap structure surrounding the active site. Only in the GPAA1^Zn^ case, the anti-correlated breathing-like motion of loops 1 and 4 is clearly recognizable in the molecular dynamics study. This observed loop dynamics opens the opportunity for speculation about its function for regulating the access to the active site for bulky substrates and/or for accompanying the catalytic process of substrate binding and release.

## Reviewers’ comments

### Reviewer’s report 1: Thomas Dandekar, MD

Minor comment: It would be at least interesting for the reader if the authors can comment (or maybe partially show) how other top programs for protein structure prediction tasks do in the hands of the authors, e.g. LOMETS or Quark. Of course, I-TASSER and MODELLER would be state of the art and are properly used by the authors for the homology modelling, but these modern heroes (as well as e.g. Rosetta or the very recent AI approach) may be help in the difficult regions of the protein.

Similarly, the authors should suggest towards discussion end what they plan next (e.g. critical validation experiemtns on their structure? Or another round of prediction modles or whatever else?).

### Reviewer’s report 2: Michael Gromiha

In this work, authors generated 3D structures of the lumenal domain of human GPAA1 including zinc ion using I-TASSER and MODELLER programs. Further, they investigated the probability of zinc binding sites and computed the interaction energies between zinc atoms and conserved residues. The manuscript is well written and the analysis has been carried out systematically. The generated models will be helpful for further studies. The following suggestions may be carried out for improvements.

1. The section, “Materials and Methods” is missing in the manuscript.
2. The RMSD between the template and modeled structure could be provided.
3. The interaction energy between zinc and amino acid residues could be given in a table.
4. It will be helpful to provide the modeled structures in a website.
5. Potential applications of the model could be discussed.

## Supplementary information

**Additional file 1: Figure S1.** Comparison of I-TASSER and MODELLER models. Superimposition of the two resulting models by I-TASSER (colored based on secondary structure) and MODELLER (grey) show the stable core folds of helices and strands of the GPAA1 that are consistent between the two models. Significant differences were observed at the highlighted loop 265–299 (dashed circle) that is absent in the M28-type enzymes and found to contain the expected additional small helix α_x_ (see Figure 1 in reference [23]). Only the I-TASSER model contains the extra helix in this region, suggesting the I-TASSER result as the better model for further analyses.

**Additional file 2: Figure S2.** Finding segments for trajectory analysis in molecular dynamics studies carried out in this work. The diagrams illustrate backbone fluctuations (RMSD) of the five Zn-binding sites of various simulation systems in this study. All simulations were carried out as triplicates (the corresponding graphs are in blue, green, and red). The trajectory portions chosen for subsequent analyses are highlighted with boxes of dashed lines.

**Additional file 3: Figure S3.** Trajectory analysis of the GPAA1^Zn^ structure. (A) We show the analysis of secondary structure of the GPAA1^Zn^ model along the molecular dynamics trajectory. The map was generated using VMD version 1.9.3 [72].. Obviously, the α/β hydrolase fold, consisting of 8 strands and 7 helices, was maintained during the simulation. (B) The distribution of the radius of gyration (Rg) along the same molecular dynamics trajectory is shown. Indeed, the model structure has remained compact.

**Additional file 4: Figure S4.** Energy contributions to zinc ion binding in the presence and absence of model substrates. Contribution of van der Waals, electrostatics, and polar solvation potentials into the Zn-binding energy of GPAA1^Zn^ at the five residue sites in the absence and presence of model substrates (PEP or SUB1).

**Additional file 5: Figure S5.** Comparison of our model structures with those of Gamage et al. [14] Superimposition of the GPAA1 model (colored based on the secondary structure) onto two GAA1 models (derived from 1RTQ using Rosetta (black) and from 4F9U using RaptorX (light gray) by Gamage et al. [14]). For better visualization, only loops of the GPAA1 model are shown (in magenta) and the loops of both models from reference [14] are hidden and presented by dashes. The additional small helix α_x_ is highlighted in black circle. The extra helix in the Rosetta model published by Gamage et al. is shown in green. The potential Zn ion position as in our GPAA1^Zn^ model (there is no zinc positioning in the models of Gamage et al.) is illustrated as the blue sphere to locate the Zn-binding cleft.

**Additional file 6: Table S1.** Summary of known M28 family 3D structures. Summary of 145 structures of M28 protein family, including 2 Zn-bound aminopeptidase and carboxypeptidase, and 1 Zn-bound cyclotransferase structures, tabulating the canonical residues forming the zinc ion and substrate binding sites.

**Additional file 7: File S1.** GPAA1_Zn_PEP.pdb - atomic structure coordinates for GPAA1^Zn^.

**Additional file 8: File S2.** GPAA1_Zn1Zn2_PEP.pdb - atomic structure coordinates for GPAA1^Zn1Zn2^.

## Data Availability

All supporting data are submitted in Supplementary Materials.
