# Supplementary figures and images for: Structural modelling of the lumenal domain of human GPAA1, the metallo-peptide synthetase subunit of the transamidase complex, reveals zinc-binding mode and two flaps surrounding the active site
Source: Biol Direct. 2020 Sep 29;15:14. doi: 10.1186/s13062-020-00266-3 (PMC7522609; doi:10.1186/s13062-020-00266-3)

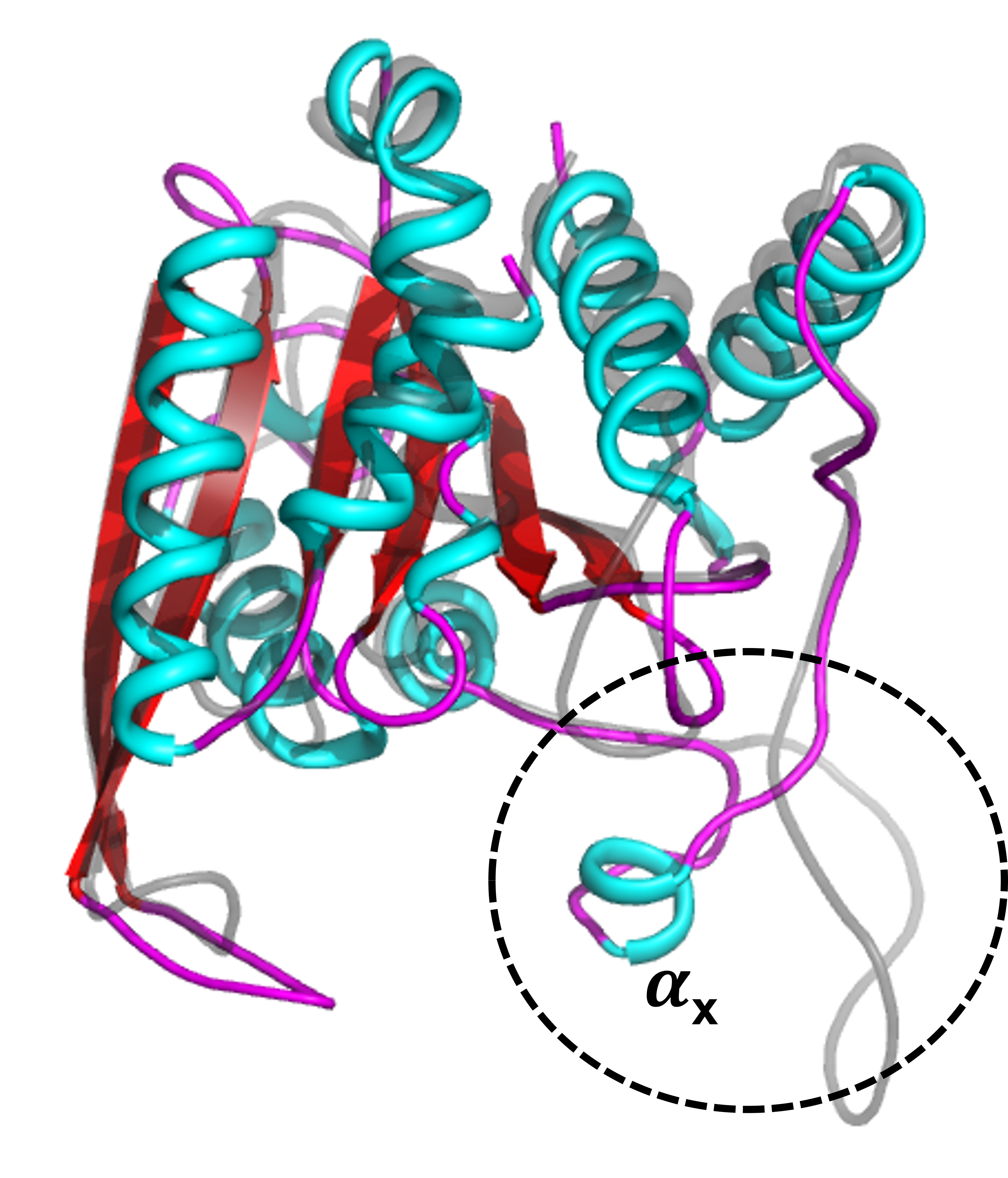

Supplement: Supplementary file 1 — Additional file 1: Figure S1. Comparison of I-TASSER and MODELLER models. Superimposition of the two resulting models by I-TASSER (colored based on secondary structure) and MODELLER (grey) show the stable core folds of helices and strands of the GPAA1 that are consistent between the two models. Significant differences were observed at the highlighted loop 265–299 (dashed circle) that is absent in the M28-type enzymes and found to contain the expected additional small helix αx (see Figure 1 in reference [23]). Only the I-TASSER model contains the extra helix in this region, suggesting the I-TASSER result as the better model for further analyses. [file 13062_2020_266_MOESM1_ESM.tiff]

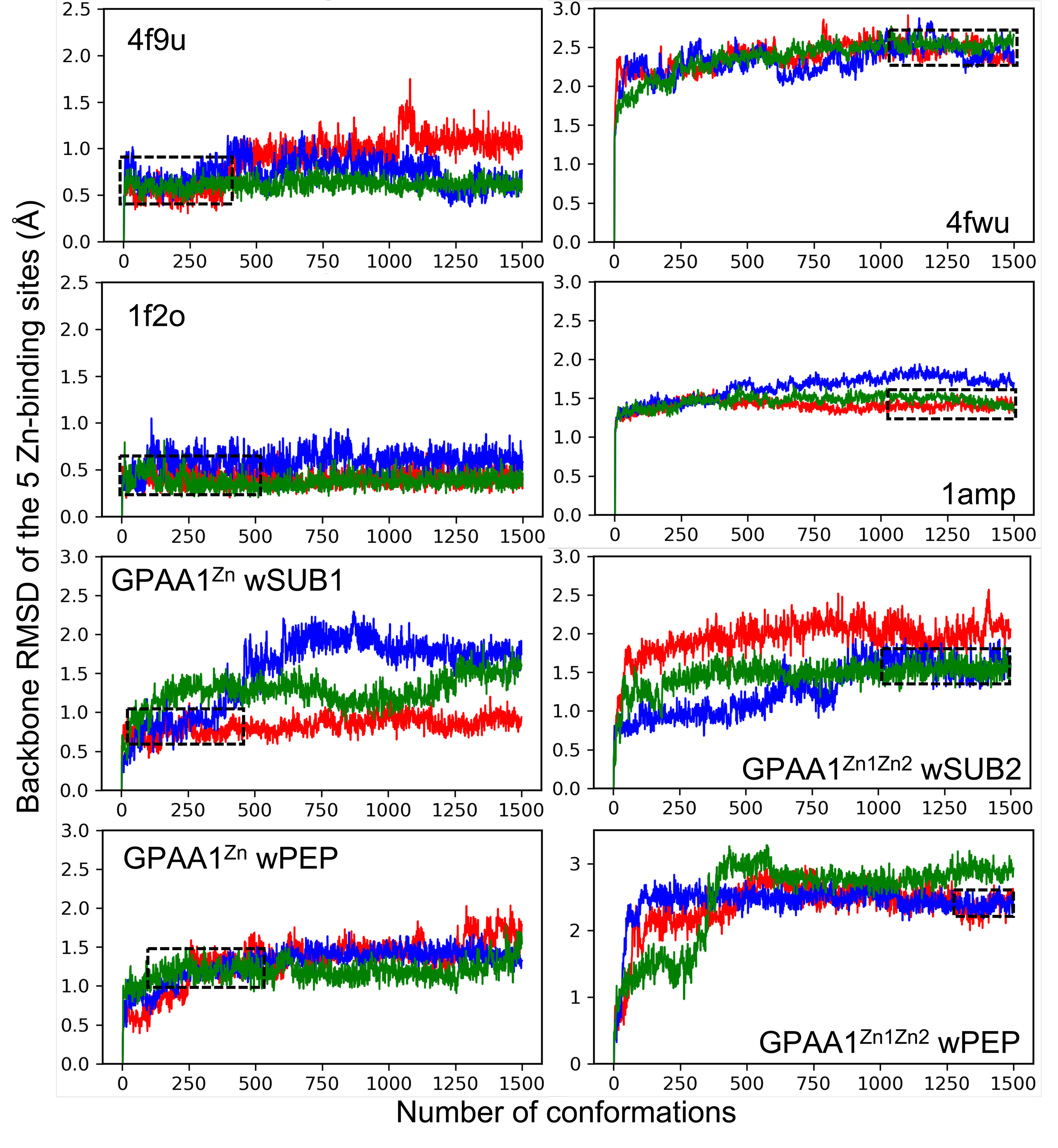

Supplement: Supplementary file 2 — Additional file 2: Figure S2. Finding segments for trajectory analysis in molecular dynamics studies carried out in this work. The diagrams illustrate backbone fluctuations (RMSD) of the five Zn-binding sites of various simulation systems in this study. All simulations were carried out as triplicates (the corresponding graphs are in blue, green, and red). The trajectory portions chosen for subsequent analyses are highlighted with boxes of dashed lines. [file 13062_2020_266_MOESM2_ESM.tiff]

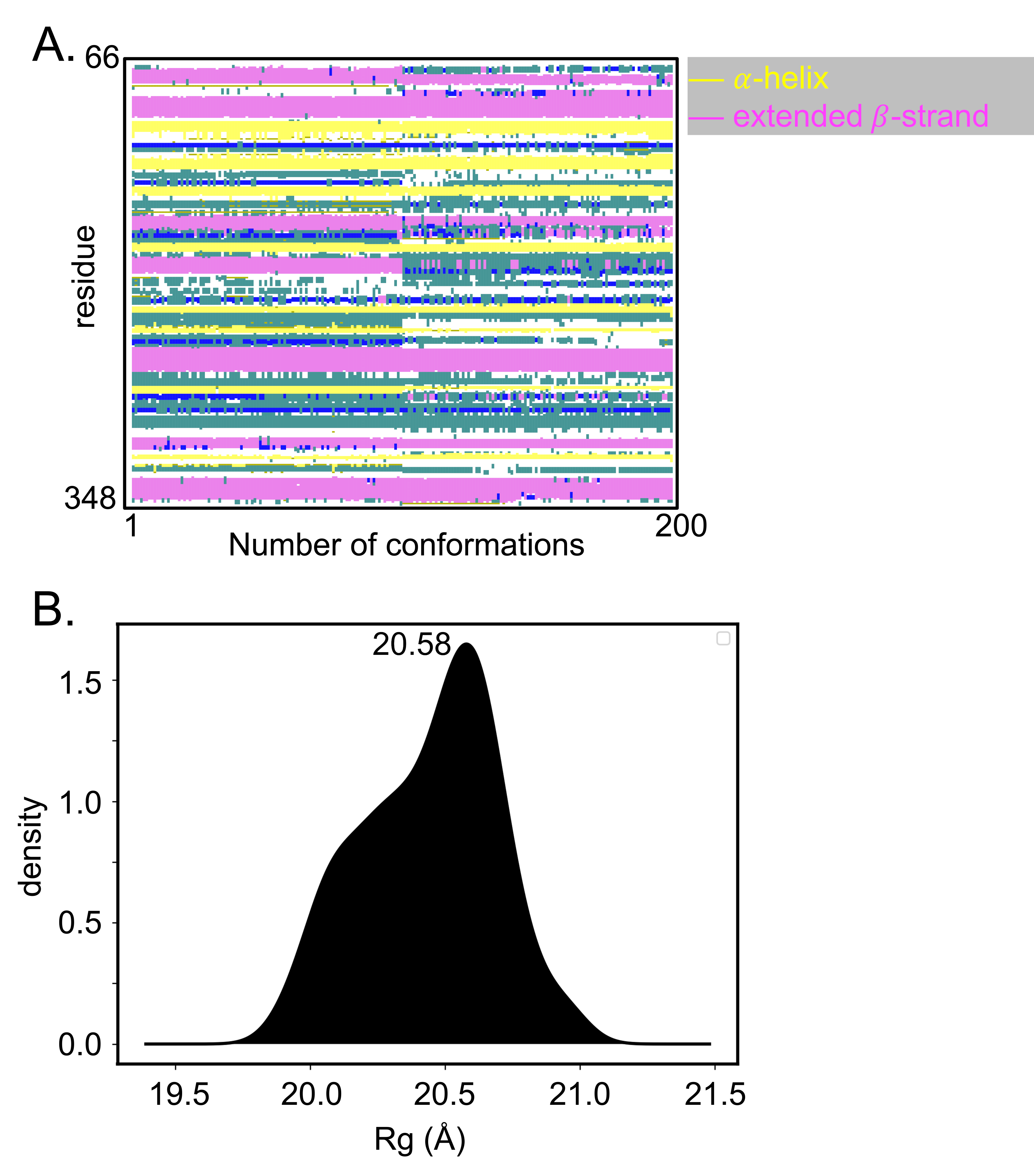

Supplement: Supplementary file 3 — Additional file 3: Figure S3. Trajectory analysis of the GPAA1Zn structure. (A) We show the analysis of secondary structure of the GPAA1Zn model along the molecular dynamics trajectory. The map was generated using VMD version 1.9.3 [72].. Obviously, the α/β hydrolase fold, consisting of 8 strands and 7 helices, was maintained during the simulation. (B) The distribution of the radius of gyration (Rg) along the same molecular dynamics trajectory is shown. Indeed, the model structure has remained compact. [file 13062_2020_266_MOESM3_ESM.tiff]

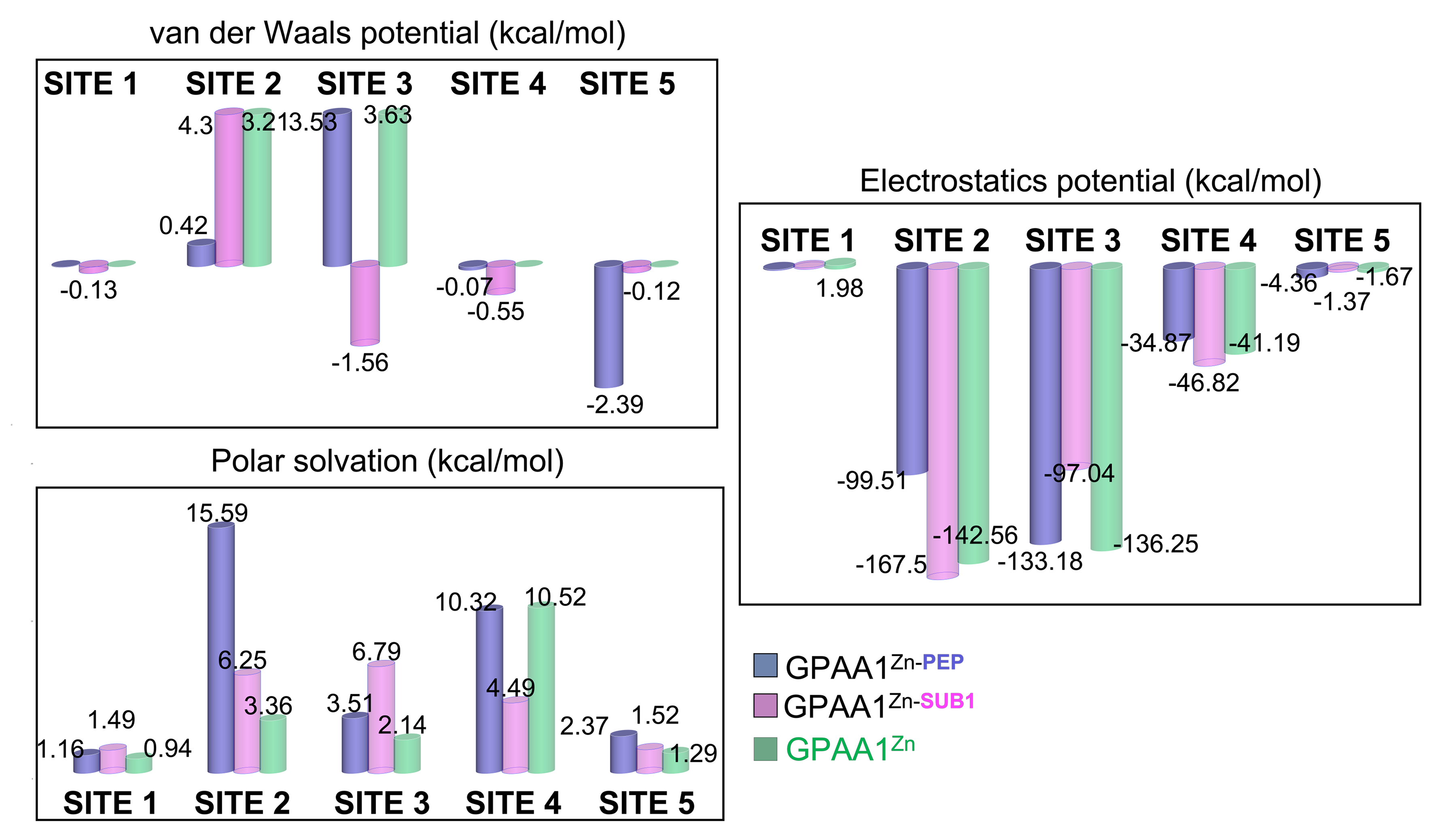

Supplement: Supplementary file 4 — Additional file 4: Figure S4. Energy contributions to zinc ion binding in the presence and absence of model substrates. Contribution of van der Waals, electrostatics, and polar solvation potentials into the Zn-binding energy of GPAA1Zn at the five residue sites in the absence and presence of model substrates (PEP or SUB1). [file 13062_2020_266_MOESM4_ESM.tiff]

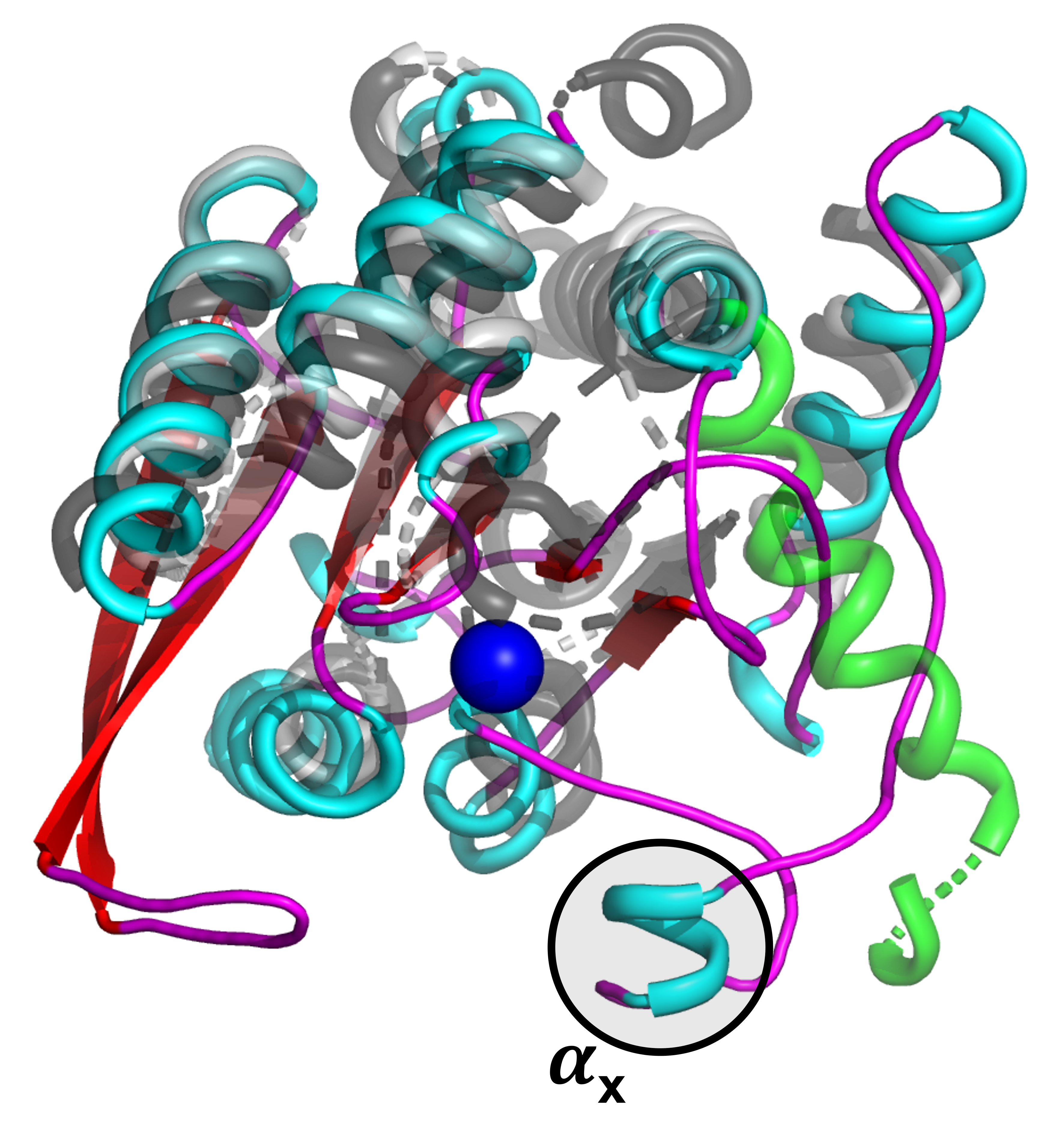

Supplement: Supplementary file 5 — Additional file 5: Figure S5. Comparison of our model structures with those of Gamage et al. [14] Superimposition of the GPAA1 model (colored based on the secondary structure) onto two GAA1 models (derived from 1RTQ using Rosetta (black) and from 4F9U using RaptorX (light gray) by Gamage et al. [14]). For better visualization, only loops of the GPAA1 model are shown (in magenta) and the loops of both models from reference [14] are hidden and presented by dashes. The additional small helix αx is highlighted in black circle. The extra helix in the Rosetta model published by Gamage et al. is shown in green. The potential Zn ion position as in our GPAA1Zn model (there is no zinc positioning in the models of Gamage et al.) is illustrated as the blue sphere to locate the Zn-binding cleft. [file 13062_2020_266_MOESM5_ESM.tiff]
